# Development of a cost of illness inventory questionnaire for children with autism spectrum disorder in South Asia

**DOI:** 10.1186/s12913-022-08508-y

**Published:** 2022-09-08

**Authors:** Divya Chaudhary, Bhargav Bhat, Gemma E. Shields, Linda M. Davies, Jonathan Green, Tara Verghis, Reetabrata Roy, Divya Kumar, Minal Kakra, Vivek Vajaratkar, Gitanjali Lall, Sonakshi Pandey, Sanchita Johri, Saani Shakeel, Vikram Patel, Monica Juneja, Sheffali Gulati, Gauri Divan

**Affiliations:** 1 Sangath, House No 451 (168), Bhatkar Waddo, Socorro, Porvorim, Bardez, Goa 403501 India; 2 The University of Manchester, Manchester, UK; 3 Goa Medical College, Bambolim, Goa India; 4 Department of Global Health and Social Medicine, Harvard Medical School, Boston, USA; 5 Maulana Azad Medical College and Assoc. Lok Nayak Hospital (MAMC), New Delhi, India; 6 All India Institute of Medical Science, New Delhi, India

**Keywords:** Autism spectrum disorder, Resource use, Children, Low- and middle-income countries, Cost-of-illness, Economic evaluation

## Abstract

**Background:**

The economic burden of autism is substantial and includes a range of costs, including healthcare, education, productivity losses, informal care and respite care, among others. In India, approximately, 2 million children aged 2–9 years have autism. Given the likely substantial burden of illness and the need to identify effective and cost-effective interventions, this research aimed to produce a comprehensive cost of illness inventory (COII) suitable for children with autism in South Asia (India) to support future research.

**Methods:**

A structured and iterative design process was followed to create the COII, including literature reviews, interviews with caregivers, pilot testing and translation. Across the development of the COII, thirty-two families were involved in the design and piloting of the tool. The COII was forward translated (from English to Hindi) and back translated. Each stage of the process of development of the COII resulted in the further refinement of the tool.

**Results:**

Domains covered in the final COII include education, childcare, relocation, healthcare contacts (outpatient, inpatient, medical emergencies, investigations and medication), religious retreats and rituals, specialist equipment, workshops and training, special diet, support and care, certification, occupational adjustments and government rebates/schemes. Administration and completion of the COII determined it to be feasible to complete in 35 minutes by qualified and trained researchers. The final COII is hosted by REDCap Cloud and is a bilingual instrument (Hindi and English).

**Conclusions:**

The COII was developed using experiences gathered from an iterative process in a metropolitan area within the context of one low- and middle-income country (LMIC) setting, India. Compared to COII tools used for children with autism in high-income country settings, additional domains were required, such as complimentary medication (e.g. religious retreats and homeopathy). The COII will allow future research to quantify the cost of illness of autism in India from a broad perspective and will support relevant economic evaluations. Understanding the process of developing the questionnaire will help researchers working in LMICs needing to adapt the current COII or developing similar questionnaires.

**Supplementary Information:**

The online version contains supplementary material available at 10.1186/s12913-022-08508-y.

## Background

Autism Spectrum Disorder (ASD) or autism is a lifelong neurodevelopmental condition, typically diagnosed in early childhood, with symptoms including impairments in social interaction and communication and the presence of repetitive patterns of behaviour, interests and/or activities [1]. The global prevalence rates for autism are 1 in 160 children [2] accounting for 121 Disability-Adjusted Life-Years (DALYs) per 100,000 of the population [3]. In India a study for nine neurodevelopmental disorders found a prevalence of approximately 1% [4], resulting in more than 2 million young children with autism requiring care. Autism is associated with substantial care needs, both for the individual and their families, posing challenges in education, employment and independent living [5]. Another significant impact on families is the increased stress, both emotional and financial [6]. Co-occurring conditions (including epilepsy, physical health problems and psychiatric disorders) also add to the increased health needs of this population [7].

A global review of the economic costs of autism found that individuals, or families with children with autism, are likely to experience multi-faceted financial burdens and higher costs, relative to other individuals or families with children [8]. This included the cost of healthcare (including therapies), education, productivity losses, informal care, accommodation and respite care. The review highlighted the difficulty of measuring some costs, which may potentially underestimate costs and result in inaccurate estimates of the societal cost burden of autism. Comparisons across countries were limited due to differences in service provision, data sets and heterogeneity in populations. Additionally, all but one (from China, a middle-income country) of the included studies used data from high-income countries, whereas most children with autism live in low- and middle-income countries (LMICs) [9].

There is limited evidence on the economic costs of autism in South Asia. A recent study conducted in the state of Odisha, India, which has a per capita earning of US $476.44 (INR 24,275.00), found that treatment expenditure for children with autism ranged from US $16.49 (INR 1000) to US $82.49 (INR 5000) per month excluding travel and other indirect costs [10]. Media sources have highlighted the burden faced by families in metropolitan areas, citing expenditures up to US $462.89 (INR 30,000 per month) on therapies in a system with no health insurance coverage for developmental conditions [11, 12].

The Communication-centred Parent-mediated treatment for Autism Spectrum Disorder (ASD) in South Asia (COMPASS) project is conducting a randomized controlled trial, to evaluate the effectiveness and cost-effectiveness of a parent-mediated intervention for autism in New Delhi, India [ISRCTN ID: 21454676] [13]. A key issue identified in the development of the economic component of the trial was the lack of a comprehensive and culturally relevant instrument to collect service use data to estimate costs. Furthermore, there is a lack of evidence on the cost burden associated with ASD in South Asia. Cost of illness (COI) studies are economic studies that aim to quantify the costs (economic burden) related to a particular disease or condition [14, 15]. These can be useful in informing research and funding priorities. Subsequently, we developed a COI questionnaire. The aim was to design a questionnaire that would provide an accurate understanding of the costs of ASD in India and capture the costs relevant for economic evaluation, from a societal perspective.

This paper describes the development of this new tool (the COMPASS Cost of Illness Inventory [COII]), with an overview of the steps taken to develop the tool and key areas that were considered prior to finalizing the tool. This process will have relevance across other LMICs and for other complex disorders.

## Methods

**Fig. 1:**
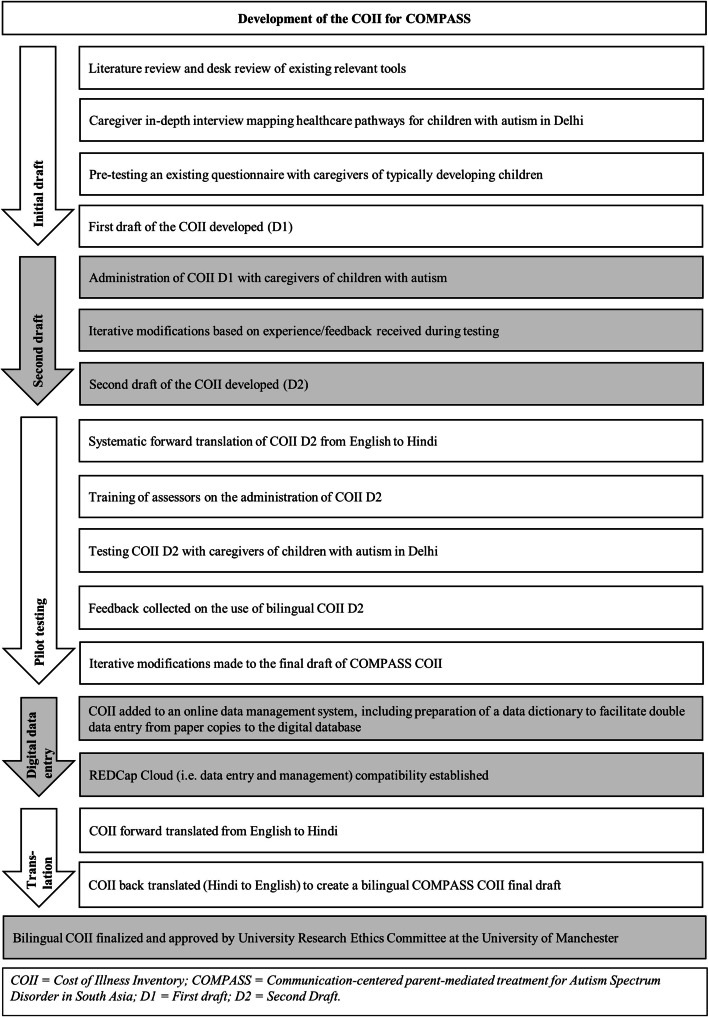
Flow chart of the COMPASS COII development process

The study used mixed methods to design, revise and finalize the COII. An overview of the methods and design phases is provided in Fig. 1.

Across the phases of work, qualitative methods were a key component of the development and testing work and are described below, with details specific to each study phase reported more in later sections. Qualitative work was conducted from November 2018 to October 2019, in New Delhi, India. The study invited families, purposively sampled to include those with a varied experience of the health system, to the initial development of the tool. Participants were families with an autistic child under the age of 11 years in New Delhi and were referred by the COMPASS trial collaborating sites (All India Institute of Medical Science (AIIMS) and Maulana Azad Medical College and assoc. Lok Nayak Hospital (MAMC), New Delhi). These are tertiary health care providers within child development services, servicing a diverse range of families in the National Capital Region of India. Appointments were arranged telephonically by researchers explaining the purpose of the engagement. Informed consent was obtained by researchers, at the home of families. Caregivers were informed of the purpose of interviews and were encouraged to provide feedback on all aspects of the interview and questionnaires. All interviews were audio recorded and transcribed by a bilingual researcher. Qualitative interview analysis was conducted using a uniform thematic grid format.

### Development of initial draft

**Fig. 2:**
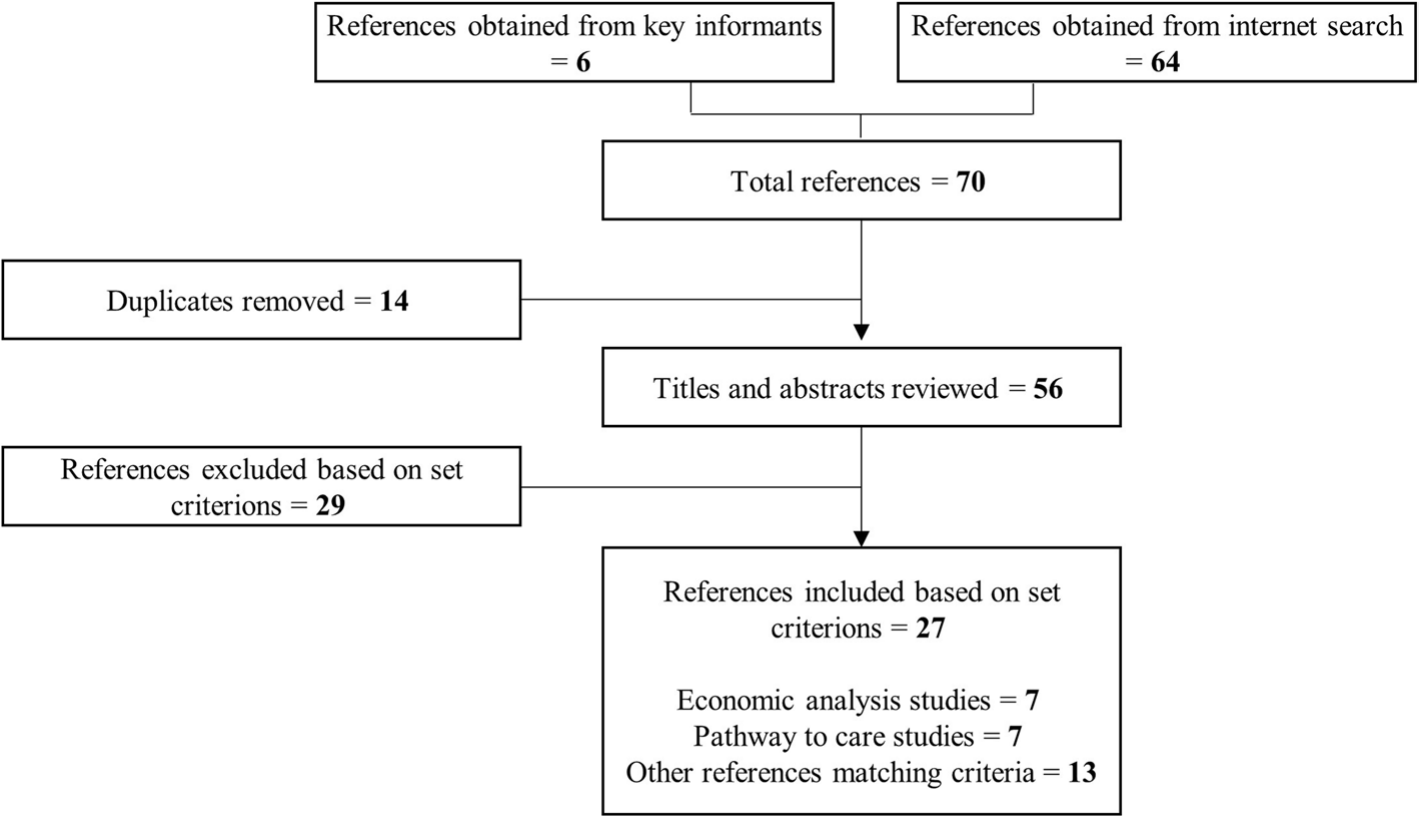
Literature review process

A literature review was conducted from December 2018 to March 2019 to identify existing relevant service use and cost data collection tools, which could inform initial items to include in the COII. PubMed, InMed and Google Scholar were searched to identify publications from 2003 to 2019. Search terms used included condition specific terms (e.g. “autism” and “ASD”), study design terms (e.g. “economic evaluation” and “cost of illness”) and a term to identify studies in India (i.e. “India”). Inclusion criterion included economic evaluations for autism, cost or burden of illness studies for autism and pathways to care studies in low and middle-income countries with a focus on autism. A researcher (DC) conducted the initial search with the assistance of a second researcher (BB). To supplement searches, the project leads suggested key informants who were approached to support the identification relevant publications to inform the questionnaire development. Searching was followed by a review of titles and abstracts and the removal of duplicates and publications which did not meet inclusion criterion, ending with a detailed reading of shortlisted articles (Fig. 2).

In parallel, in-depth interviews were carried out to understand the help-seeking behaviours of families with fifteen caregivers of children with autism. These interviews were conducted by bilingual researchers (DC, DK and MK) who used a semi-structured questionnaire. The semi-structured guide explored the age of recognition of autistic symptoms, pathways to care for families, the age of diagnosis, health system interactions, varieties of practitioner interactions, costs of services as well as the respondent’s opinions on these interactions. These interviews were conducted with 11 families of children with autism and with a further 4 families of typically developing children along with the administration of the PASS Plus (Parent-mediated Intervention for Autism Spectrum Disorders in South Asia Plus) COII, (an unpublished tool used by the research team in a rural context). The children’s age ranged from 5 to 11 years and the respondents included mothers (*n* = 6), fathers (*n* = 5), both parents (*n* = 3) and one set of grandparents. All interviews were conducted in Hindi and transcribed into English. A line by line analysis was conducted using the framework analysis method [16] to identify themes which included time and monetary costs of help seeking (outpatient and inpatient), investigations, emergencies, religious trips/ events, medications and special equipment. Initial codes and themes were reviewed by more than one team member (DC, VV, GD) to minimize researcher bias. This opportunity allowed the evaluation of the instrument language and design, time needed for completion, ease of administration, as well as it’s applicability to an urban setting. The aim was to map care seeking pathways for families, which included diagnostic, treatment, rehabilitative and educational services.

The results of the literature review, desktop instrument review, in-depth interviews, and trial of an existing tool were used to produce the first draft of COII [D1] in English. A fluent bilingual researcher translated the document into Hindi.

### Development of the second draft

This stage of testing the COII D1 aimed at understanding questionnaire design and flow, acceptability of the language used, the ability to capture all relevant information, the recall capacity of caregivers and the time burden of administration. The COII D1 was administered with caregivers of children with autism. All participants met previous reported criteria, though the upper age limit of children was expanded to 12 years. Families were engaged as pre-trial practice cases for the COMPASS trial intervention team. Multiple rounds of interim discussions with team leads and domain experts, led to modifications of the draft. Expansions and modification resulted in COII draft two [D2], which was reviewed and approved by a member of the Trial Steering Committee with expertise in health economics.

### Pilot testing

In preparation for pilot testing, the COMPASS COII D2 was translated into Hindi (discussed in more detail below) and a bilingual version with English was prepared. The pilot phase aimed to assess the acceptability of the flow of questions, ease of data capture and the effectiveness of probes and translations, as well as identify any final amendments required. Six researchers for the COMPASS trial were trained to administer the COII D2. After completing pilot testing, assessors provided feedback on their experience of using the tool, following which refinements were made. A bilingual version of the COMPASS COII was finalised and ethical approval to administer the tool within the COMPASS trial was obtained.

### Digital data entry

Once the COII was finalised as described above, structural changes, such as the placement of tables within the questionnaire and variable names, were created to make it compatible with REDCap Cloud (RCC), the data capture and management platform used in the COMPASS trial. To facilitate double data entry from the paper version to the RCC digital data platform, a Case Report Form (CRF) was prepared on RCC for COMPASS COII. This process involved generating a data dictionary of 952 possible responses that were incorporated into the RCC. These responses where derived from the experiences of the piloting work. Consistency checks on RCC were validated by running multiple rounds of examinations based on pilot interviews by the data management team.

### Translation

Prior to pilot testing the COMPASS COII D2 was forward translated from English to Hindi by researchers who were fluent in both languages; developing a bilingual version. The Hindi translation of COMPASS COII D2 was revised based on the pilot testing experience to ensure translation equivalence parameters (semantic, content and technical equivalence) [17]. This version was then back translated by an external consultant, resulting in a final version of the COII.

## Results

The final COII tool is published on the Database of Instruments for Resource Use Measurement (DIRUM) and is available to use free of charge with permission (http://www.dirum.org/instruments/details/116). The final tool is also included in the supplementary material.

### Literature review

Figure 2 reports details of the number of papers identified, screened and selected for full review to inform the COII development. Twenty-seven full text articles were included in the review. References of the identified studies are included in the supplementary material.

Discussions on identified publications allowed the identification of three key existing tools: the CA-SUS used in a UK autism trial [18], the CSRI used in India in a mental health treatment evaluation [19] and the unpublished PASS Plus COII, used in a pilot study leading up to the COMPASS trial [20]. The questionnaire used in the PASS Plus project [unpublished], being the most contextually appropriate, was used with a sample of families to develop the COII D1.

### Qualitative interview and testing feedback

Thirty-two participant families were involved in the qualitative explorations conducted during the design and piloting of the tool, with their characteristics reported in Table 1.

**Table 1:** Background characteristics of families involved in COMPASS COII development process

**Caregiver type**	*n* (%)	
Caregiver of a child with autism	28 (88%)	
Caregiver of a typically developing child	4 (14%)	
**Gender of child**		
Female	6 (19%)	
Male	26 (71%)	
**Key respondents**		
Mother	17 (54%)	
Father	10 (31%)	
Both parents	3 (9%)	
Both grandparent’s	1 (3%)	
Grandmother	1 (3%)	
**Family structure**		
Joint	13 (41%)	
Nuclear	19 (59%)	
Mean age of child at time of interview in months (SD)	84.75 (31.34)	
**Employment**	Mother, *n* (%)	Father, *n* (%)
Homemaker	25 (79%)	0 (0%)
Junior officer/mid-level officer/senior officer	2 (6%)	16 (50%)
Self employed	1 (3%)	8 (25%)
Clerical	0 (0%)	2 (6%)
Part time job	3 (9%)	0 (0%)
Skilled worker	0 (0%)	1 (3%)
Unskilled worker	1 (3%)	4 (13%)
Unemployed	0 (0%)	1 (3%)

The in-depth and testing interviews identified a range of hidden or contextually specific costs, which were not identified in the literature review (e.g. religious trips). This reflected the need for tools to be culturally specific and to reflect help seeking in an urban Indian health system.

### Domains/cost categories included in the final tool

The final domains included in the tool reported in Table 2. Note that healthcare services (outpatient and inpatient contacts) aim to capture both ASD specific visits and wider visits (e.g. for physical health). The COII also allows for differentiation by provider, i.e. private versus government. The final list of domains demonstrates the wide range of costs that should be considered when quantifying the economic burden of ASD and highlights how focusing solely on healthcare use may underestimate the burden.

**Table 2:** Domains addressed in the final COII

Item No.	Domain	Description	Cost category		
			Direct medical	Direct non-medical	Indirect^**a**^
1.	Education	Monetary and time resources used in providing the child with educational services from private and public providers.		**✓**	**✓**
2.	Childcare	Monetary and time resources used by the caregivers to care for children whilst they undertake other personal and professional commitments.		**✓**	
3.	Outpatient contacts	Monetary and time resources used by caregivers to access various healthcare and rehabilitative services for the child’s autism in a hospital setting. Monetary expenses in terms of consultation fees, travel and food. Time expenses in terms of travel, waiting and time spent with service providers for each consultation.	**✓**	**✓**	**✓**
4.	Inpatient contacts	Monetary costs incurred by for hospital admissions (overnight stays), including any associated investigations, medications, etc. The time commitment is also captured broadly based on number of nights accompanied by a caregiver.	**✓**		**✓**
5.	Relocation	Monetary costs incurred by caregiver in relocating their residence because of child’s autism due to a variety of reasons e.g. to be nearer services, societal stigma, lack of family support related to the child’s autism.		**✓**	
6.	Emergencies/ accidents	Monetary costs related to medical emergencies related to the child.	**✓**		
7.	Religious trips, retreats and rituals	Many caregivers believed in the healing nature of undertaking religious rituals and visits which can incur monetary costs. Time expenses are collected only for extensive commitments, e.g. pilgrimages.		**✓**	
8.	Investigations	Monetary costs related to multiple investigations for child with autism, especially at early stages of diagnosis to confirm the child’s autism.	**✓**		
9.	Complimentary medication	Monetary cost of supplements recommended by specialists to help the child’s development from a nutritional perspective.	**✓**		
10.	Medication	Monetary costs associated with medications recommended by specialists to help the child’s co morbidities or other illness.	**✓**		
11.	Equipment	Monetary costs associated with equipment recommended by specialists to support child’s development or part of some treatment routine.		**✓**	
12.	Workshops and training	Caregivers spend time and money attending seminars or trainings related to autism, to develop their knowledge and skills to support caregiving. Monetary and time expenses collected.		**✓**	
13.	Special diet	Monetary costs associated with prescribed special diets. Expenses in terms of time can also be included.		**✓**	**✓**
14.	Support and care	Monetary costs related to loss of income for leave taken to support child’s autistic needs and the loss of allotted or paid leaves for the individual which could have been used in other situations e.g. vacation (for leisure). This includes leave taken for child’s hospitalization (inpatient contacts).			**✓**
15.	Cost of certification	Disability certificates are free and provide the caregiver the ability to benefit from certain rebates and schemes from the government targeted towards caregivers taking care of disabled individuals. Time costs associated to getting this certification can be identified.		**✓**	
16.	Occupational adjustments	Caregiver’s may change their employment status to become full time caregivers. The monetary costs related to this can be included, using documentation of the last salary drawn and the year.			**✓**
17.	Government rebates/schemes	The union government of India provides rebates for caregivers with dependents with disabilities, details of which can be collected in this section. Other schemes by public institutions help in reducing the caregiver’s overall monetary burden.			

*Notes*: ^a^ Note that indirect costs pertain to losses in the productivity of caregivers (e.g. time spent accompanying a child to visits, days missed from work). Time is reported in minutes and subsequently the human-capital method could be used for costing

COI studies and economic evaluations can take different perspectives; most commonly that of the healthcare system or a broader societal perspective [14, 21] (refs). Costs related to the domains of the COII fall under three key categories; direct medical costs, direct non-medical and indirect costs (productivity losses) [14]. Table 2 presents the type of cost that be ascertained using each domain (assuming relevant unit costs can be identified). Whilst the COII was developed to suit a potential societal perspective (i.e. incorporating indirect costs), it can be easily adapted to restrict to narrower perspectives.

### Structure of the tool

Due to the nature of the information being collected and the wide range of answers that can be generated, the COII has been designed to allow a flexibility in recording information of various kinds. For example, closed responses in the form of codes, binary yes/no, numeric values with different units (e.g. year, days, minutes, costs, etc.), as well as free text. The form allows the assessor to move between sections of the questionnaire based on the flow of conversation with the caregiver that improves the ease of administration. A free text box allows the recording of notes and the experience of administration from the assessor’s perspective, and the documentation of any unusual caregiver experiences not adequately captured in the sections of the form. As an example, it allows mapping of the numerous visits required to get a disability certificate. Another instance during piloting was of a father who began night shifts, to be a full-time caregiver while the mother worked during the day. These qualitative observations can help in complimenting the quantitative data generated by the instrument.

### Feasibility and burden of administration

The administration of the COMPASS COII was feasible in all pilot households and took an average of 35 minutes to complete. An adjustment made to the questionnaire aimed at reducing the time for administration is an initial introductory question for each domain; allowing the section to be explored or skipped based on the caregiver response. Questions about medications and other products used are limited to the previous 6 months, to minimise recall bias.

## Discussion

Cost of illness studies and economic evaluations for lifelong neurodevelopmental conditions like autism have to date mostly been conducted within high-income settings. However, most of the global population of children with autism live in LMICs and evidence from high-income countries is not generalisable due to the vast differences across contexts. In this paper, we report the development of a COII which aims to provide researchers with a tool to get an accurate understanding of the costs of ASD in a LMIC setting. The design of this tool accounts for its use in a complex setting, where costs related to health are likely to be varied and complicated. The development process has highlighted the complexities of the lives of families of young children with autism on their care seeking journeys and will allow an accurate estimate of the cost of illness related to autism in this context in South Asia. The current design of the tool should be able to capture costs related to autism and co-occurring conditions. Though this process was time and resource intensive, the questionnaire has been designed to be able to reflect multiple payer and provider perspectives which exist in many low resource settings.

Our experience illustrates that a standard COII from one setting cannot simply be applied in another (especially when moving between countries with different income levels); but will require comprehensive adaptations to reflect contextual aspects of the healthcare systems (private and public), socio demographic and economic distributions within a population. We see this development process as a unique first of its kind attempt to map the multi-sectorial costs affecting families of young children with autism in an LMIC. We also consider that it can be expanded to support research to assess costs of other neurodevelopmental disabilities, but also to understand the lifetime costs of autism in LMIC.

The strengths of this tool include its ability to record complex perspectives, for example, personal transport costs to visit subsidised or free public health services amounted to a significant expense for many families. Similarly, the questionnaire allows us to document relocation costs of moving to an area which is closer to autism services. The questionnaire also separates out inpatient and outpatient care costs, since these have significantly different components (e.g. travel, stay and subsistence); and the support that some caregivers employment insurance allow. It has also been designed keeping in mind usability without compromising its primary objective of collecting relevant and accurate information. The bilingual version of the questionnaire allows the tool to accommodate the use of English for many technical aspects of services by caregivers who primarily speak in the local language. Being RCC compatible further aids data accuracy and streamlines the process for data analysis. Costs can be compartmentalised to inform policy, and to generate information which will allow an understanding of the economic burden of autism to families and add value to a very limited evidence base. By providing clear introductory scripts and probes for different sections and an accompanying code sheet, it ensures that information is captured uniformly. It does not require extensive training and can be administered in clinical or community settings. Current guidelines recommend that economic evaluations alongside clinical trials prioritise high-cost resources and those that are expected to differ between treatment arms [22]. This COII will allow researchers in this setting to understand what these key resources are, which may reduce the burden of data collection in future economic evaluations conducted alongside trials. Note that the COII will allow us to collect resource use and many costs, however some unit costs will need to be identified from the wider literature and resources to calculate total cost. For example, unit costs associated with government funded healthcare [23].

To the author’s knowledge, this is the first publication that reports the development of a resource use questionnaire/COII for use in LMICs. The COII questionnaire design also would allow it to be adapted to other neurodevelopmental disabilities, or other LMIC settings, and can be used as a starting point by other researchers. Relevant domains can be included or excluded according to study perspectives. This tool can be adapted across all income settings but is particularly relevant to LMIC, and will allow studies to collect comparable data across contexts, a critical missing area in autism research. Note that this paper describes the development of the tool only, the validity of the COII will be explored in a future study using evidence from a trial cohort.

The limitations of this tool include its current focus on children, which does not reflect the nature of autism as a lifelong condition, and the need to explore the costs of autism across the lifespan. There are some areas of costs to families which do not map into any of the current areas of the questionnaire, though they may be substantial (e.g. searching for an inclusive school). These are likely to be challenging and potentially unfeasible to collect. Another limitation is caregiver recall and knowledge, particularly around the nature of the specialist visited (e.g. not being able to differentiate a therapist from a doctor, which in a private sector may have different cost implications). Recall is a known challenge related to resource use measurement and further research could help to investigate whether carer recall is reliable (e.g. by comparing electronic hospital data versus carer report) [24]. However, many of the domains included in the COII will only be available through the carer and so this problem is unavoidable. Furthermore, careful consideration of the follow-up period and assessment points can help to reduce recall bias whilst minimising participant burden [25, 26]. Studies have investigated reliability in self-report resource use questionnaires across a range of conditions (e.g. cancer, bowel disease and psychiatry), but to the authors knowledge there are no such studies investigating parents ability to recall children’s resource use [27–30]. This could be investigated in future research projects. Since the development of this tool was nested in an ongoing trial based in an urban Indian setting, there is a possibility that contextually specific costs in rural settings may not be represented. The tool aimed to allow researchers to collect sufficient data to allow for a societal perspective, however there may be further challenges in doing this robustly. For example, in this population productivity losses (and informal care costs) are related to caregivers of children (informal carers) with autism but as the children are young, it would be expected that a portion of this productivity loss would be incurred even if the children did not have autism. Further validation of the tool will be useful, though validation of resource and cost questionnaires is challenging [24]. A larger sample will complete the COII as part of the COMPASS trial which will allow us to assess the completeness of data collection. Finally, it is important to note the limitations and debate around COI studies. For example, highlighting a high cost of illness does not necessarily imply that cost savings are needed (i.e. it does not evidence an inefficient allocation of resources and the burden of a disease extends far beyond costs (e.g. to patient and carer outcomes) [15]. It should also be noted that COI studies often place an emphasis on how cost savings could be achieved if a condition were eradicated, however, this is not the aim or intention of our tool [14]. Using the tool as part of an economic evaluation may be more useful, as it will allow researchers to investigate changes in cost as a result of intervention. We propose this tool to support COI studies and economic evaluations in a relevant research context. However, researchers will likely need to adapt the tool to meet their specific research questions and planned methodologies.

## Conclusions

The work demonstrates how an iterative process, building on the existing evidence base, and applying qualitative research techniques can help to produce a comprehensive and culturally relevant Cost of Illness Inventory that is acceptable and feasible to use in a complex setting across different perspectives. When compared with equivalent tools used in high-income countries, it was found that additional domains were needed to ensure that cost data were extensive, for example the tool can collect costs associated with religious activities and complementary health practices. This provides a systematic example to other researchers in LMIC settings who are developing similar questionnaires. More importantly it will allow future research to accurately quantify the cost of illness of ASD in India, simultaneously allowing the collection of data in India and other LMICs which can be comparable across contexts to support economic evaluations along with critical data to help scale up services to vulnerable families.

## Supplementary Information

**Additional file 1: Supplementary material**.

## Data Availability

The final COII is freely available with written permission from the authors (https://www.dirum.org/instruments/details/116).

## Abbreviations

ASD: Autism Spectrum Disorder
CA SUS: Child and Adolescent Service Use Survey
COI: Cost of illness
COII: Cost of Illness inventory
COMPASS: Communication-centered parent-mediated treatment for Autism Spectrum Disorder in South Asia
CRF: Case Report Form
CSRI: Client Service Receipt Inventory Form
DALY: Disability Adjusted Life Year
DIRUM: Database of Instruments for Resource Use Measurement
LMIC: Low and Middle Income Countries
PACT: Pediatric Autism Communication Trial
PASS Plus: Parent-mediated Intervention for Autism Spectrum Disorders in South Asia Plus
PREMIUM: Program for Effective Mental health Interventions in Under-resourced health systems
RCC: REDCap Cloud
UREC: University Research Ethics Committee
