# Supplementary material for: Development of a cost of illness inventory questionnaire for children with autism spectrum disorder in South Asia
Source: BMC Health Serv Res. 2022 Sep 8;22:1137. doi: 10.1186/s12913-022-08508-y (PMC9461098; doi:10.1186/s12913-022-08508-y)
Supplement: Supplementary file 1 — Additional file 1: Supplementary material. [file 12913_2022_8508_MOESM1_ESM.pdf]

Development of a cost of illness inventory questionnaire for children with autism spectrum disorder in South Asia

Corresponding author: Gauri Divan (gauri.divan@sangath.in)

Supplementary material

Literature search results

Economic Evaluation Studies	
1.	Sharma P, Das SK, Deshpande SN. An estimate of the monthly cost of two major mental disorders in an Indian metropolis. Indian J Psychiatry. 2006;48(3):143–8.
2.	Byford S, Cary M, Barrett B, Aldred CR, Charman T, Howlin P, et al. Cost-effectiveness analysis of a communication-focused therapy for pre-school children with autism: results from a randomised controlled trial. BMC psychiatry. 2015;15:316-.
3.	Buescher AVS, Cidav Z, Knapp M, Mandell DS. Costs of Autism Spectrum Disorders in the United Kingdom and the United States. JAMA Pediatr. 2014 Aug 1;168(8):721–8.
4.	Lavelle TA, Weinstein MC, Newhouse JP, Munir K, Kuhlthau KA, Prosser LA. Economic Burden of Childhood Autism Spectrum Disorders. Pediatrics. 2014 Mar 1;133(3):e520–9.
5.	Knapp M, Romeo R, Beecham J. Economic cost of autism in the UK. Autism. 2009 May;13(3):317–36.
6.	Roddy A, O'Neill C. The economic costs and its predictors for childhood autism spectrum disorders in Ireland: How is the burden distributed? Autism. 2018 Oct 1;1362361318801586.
7.	Patel V, Weobong B, Nadkarni A, Weiss HA, Anand A, Naik S, et al. The effectiveness and cost-effectiveness of lay counsellor-delivered psychological treatments for harmful and dependent drinking and moderate to severe depression in primary care in India: PREMIUM study protocol for randomized controlled trials. Trials. 2014 Apr 2;15(1):101.
Pathways to Care Studies	
1.	Chakraborty S, Kommu JVS, Srinath S, Seshadri SP, Girimaji SC. A comparative study of pathways to care for children with specific learning disability and mental retardation. Indian Journal of Psychological Medicine. 2014 Jan 1;36(1):27.
2.	Juneja M, Sairam S. Autism Spectrum Disorder - An Indian Perspective. In [cited 2018 Dec 12]. p. 15. Available from: http://www.smgebooks.com/autism/chapters/AUT-18-12.pdf
3.	Divan G, Vajaratkar V, Desai MU, Strik-Lievers L, Patel V. Challenges, Coping Strategies, and Unmet Needs of Families with a Child with Autism Spectrum Disorder in Goa, India. Autism Res. 2012 Jun;5(3):190–200
4.	Daley TC. From symptom recognition to diagnosis: children with autism in urban India. Social Science & Medicine. 2004 Apr;58(7):1323–35.
5.	Anand P, Sachdeva A, Kumar V. Pathway to care and clinical profile of children with attention-deficit hyperactivity disorder in New Delhi, India. J Family Community Med. 2018;25(2):114–9.
6.	Rudra A, Belmonte MK, Soni PK, Banerjee S, Mukerji S, Chakrabarti B. Prevalence of autism spectrum disorder and autistic symptoms in a school-based cohort of children in Kolkata, India. Autism Research. 2017;10(10):1597–605.
7.	Desai MU, Divan G, Wertz FJ, Patel V. The discovery of autism: Indian parents' experiences of caring for their child with an autism spectrum disorder. Transcult Psychiatry. 2012 Jul 1;49(3–4):613–37.
Others	
1.	McDaid D, Byford S, Sefton T. Because it's worth it: a practical guide to conducting economic evaluations in the social welfare field: Joseph Rowntree Foundation; 2003.
2.	Beecham J, Knapp M. Costing psychiatric interventions. Measuring mental health needs. 2001;2:200-24.
3.	Reinap M, Lai T, Janno S, Tamme T, Tamm M. Cost-effectiveness of mental health interventions in Estonia. Healthcare Society in Estonia. 2005.
4.	Control CfD, Prevention. Developing an effective evaluation plan: Setting the course for effective program evaluation. Atlanta, Georgia. 2011.
5.	Petrou S, Gray A. Economic evaluation alongside randomised controlled trials: design, conduct, analysis, and reporting. BMJ [Internet]. 2011 Apr 7 [cited 2018 Nov 26];342. Available from: https://www.ncbi.nlm.nih.gov/pmc/articles/PMC3230107/

6.	Lima AFB da S, Cruz LN, Polanczyk CA, Maia CRM, Lima AFB da S, Cruz LN, et al. Economic evaluation in the field of mental health: conceptual basis. Brazilian Journal of Psychiatry. 2013 Jun;35(2):186–92.
7.	Parsonage M, Fossey M. Economic evaluation of a liaison psychiatry service: Centre for Mental Health London; 2011.
8.	Lamsal R, Zwicker JD. Economic Evaluation of Interventions for Children with Neurodevelopmental Disorders: Opportunities and Challenges. Appl Health Econ Health Policy. 2017;15(6):763–72.
9.	Shearer J, McCrone P, Romeo R. Economic Evaluation of Mental Health Interventions: A Guide to Costing Approaches. PharmacoEconomics. 2016 Jul 1;34(7):651–64.
10.	Luyten J, Naci H, Knapp M. Economic evaluation of mental health interventions: an introduction to cost-utility analysis. Evidence Based Mental Health. 2016 May;19(2):49–53.
11.	Green J, Charman T, McConachie H, Aldred C, Slonims V, Howlin P, et al. Parent-mediated communication-focused treatment in children with autism (PACT): a randomised controlled trial. Lancet. 2010 Jun 19;375(9732):2152–60
12.	Divan G, Vajaratkar V, Cardozo P, Huzurbazar S, Verma M, Howarth E, et al. The Feasibility and Effectiveness of PASS Plus, A Lay Health Worker Delivered Comprehensive Intervention for Autism Spectrum Disorders: Pilot RCT in a Rural Low and Middle Income Country Setting. Autism Res. 2019;12(2):328–39.
13.	Rahman A, Divan G, Hamdani SU, Vajaratkar V, Taylor C, Leadbitter K, et al. Effectiveness of the parent-mediated intervention for children with autism spectrum disorder in south Asia in India and Pakistan (PASS): a randomised controlled trial. Lancet Psychiatry. 2016 Feb;3(2):128–36.

Final COII (English)

COMPASS

Communication-centred Parent-mediated treatment for Autism Spectrum Disorder in South Asia (COMPASS)

Cost of Illness Inventory (COII)

ENGLISH

Section A: Assessment Information		Variable Names
A.1. Participant ID	/ / /	participant_id
A.2. Research Associate Code		racode
A.3. COII completed	1. Yes (Go to A.7)<br>2. No	coii_complete
A.4. Reason COII not completed	1. Child unavailable<br>2. Caregiver unavailable<br>3. Child unwell/irritable<br>4. Caregiver unwell<br>5. Unforeseen events causing disruption<br>6. Uncomfortable responding to item(s) in instrument<br>7. Consent withdrawal - bad past experience	coii_notcompletereason
A.5. Rescheduled Date	D D / M M M / Y Y Y Y	coii_rescheduledate
A.6. Rescheduled Time	: AM / PM	coii_rescheduletime
A.7. COII - date	D D / M M M / Y Y Y Y	coii_date

Script for the Interviewer

- The purpose of this questionnaire is for us to understand the amount of time and money that you had to invest in [Child's Name].
- We are interested in all expenses including those spent on doctor's visit, hospital admissions and tests, medicines, education and caretaking for [Child's name].
- This questionnaire usually takes 15 minutes. I will be asking you questions and it would be really helpful if you could keep any medical file/ papers that you have for [Child's Name] ready so we can refer to it. You can also show me any medicines that you are giving to [Child's Name] – so that I can accurately record the names.
- I am interested in all the information you can give me which pertains to the past **6 months.**
- If you are not able to understand the questions I am happy to clarify it for you.
- We can take a break in between whenever you feel necessary.

C.1 EDUCATION

Can you tell me if [Child's Name] has used any education services over the last **6 months?** For example

Circle response	
YES	NO

If **NO** skip to **C.2**
For **YES** refer Table 1 – inquire further in Table 2

Table 1	
**Code**	**Name**
1	Mainstream Nursery/ School
2	Special Nursery/ School
3	Resource Room in Mainstream School
4	Home tutor (Specify)
5	Others (Specify)

Instruction – Mention all responses before selecting

Table 2

**S. No**		**C.1.1**	**C.1.2**	**C.1.3**	**C.1.4**
**C.1.a**	Code **(Refer Table 1)**	☐	☐	☐	☐
**C.1.b**	Name of School/ Subject *(If Others Specify)*				
**C.1.c**	**Private** Service provider 1<br>**Government** Service Provider 2<br>*(use appropriate code)*	☐	☐	☐	☐
**C.1.d**	What was the total cost in last 6 months? **(Rs.)**				
**C.1.e**	Who paid the fees? **(Refer COII Code Sheet - Table G)**	☐	☐	☐	☐
**C.1.f**	If fees paid by others specify				
**C.1.g**	How much time did [Child's Name] spend with the Service provider in a day? (Include travel time) **(Hours : Minutes)**	☐☐ : ☐☐	☐☐ : ☐☐	☐☐ : ☐☐	☐☐ : ☐☐
**C.1.h**	How much time did you spend with [Child's Name] while he/ she was attending these services in a day? **(Hours : Minutes)**	☐☐ : ☐☐	☐☐ : ☐☐	☐☐ : ☐☐	☐☐ : ☐☐
**C.1.i**	How many days in a week did [Child's Name] attend these services?	☐	☐	☐	☐
**C.1.j**	How many months did [Child's Name] attend these services in the last 6 months?	☐	☐	☐	☐

**C.1.k**	What was the travel costs involved (Both ways) per month? **(Rs.)**				
**C.1.l**	Who paid for the travel expense? **(Refer COII Code Sheet - Table G)**	☐	☐	☐	☐
**C.1.m**	If travel cost paid by others specify				

C.2 CHILDCARE

Now I will be talking about childcare services. Have you used any of these forms of childcare for [Child's name] in the last **6 months?**

Circle response	
YES	NO

If **NO** skip to **C.3**
For **YES** refer Table 3– inquire further in the Table 4

Table 3	
**Code**	**Name**
1	Part time Nanny/ maid
2	Full time Nanny/ Childminder
3	Daycare/ Crèche
4	Others (Specify)

Table 4

**S. No**		**C.2.1**	**C.2.2**	**C.2.3**	**C.2.4**
**C.2.a**	Code **(Refer Table 3)**	☐	☐	☐	☐
**C.2.b**	If any other services used specify				
**C.2.c**	**Private** Service provider 1<br>**Government** Service Provider 2<br>*(use appropriate code)*	☐	☐	☐	☐
**C.2.d**	What was the total cost in last 6 months? **(Rs.)**				
**C.2.e**	Who paid for the services? **(Refer COII Code Sheet - Table G)**	☐	☐	☐	☐
**C.2.f**	If Others specify				
**C.2.g**	How much time did [Child's Name] spend with the Service provider per day? (Include travel time) **(Hours : Minutes)**	☐☐ : ☐☐	☐☐ : ☐☐	☐☐ : ☐☐	☐☐ : ☐☐

C.2.h	How much time did you spend with [Child's Name] while he/ she was attending these services in a day? **(Hours : Minutes)**	:	:	:	:
C.2.i	How many days in a week did [Child's Name] attend these services?				
C.2.j	How many months did [Child's Name] attend these services in the last 6 months?				
C.2.k	What was the travel costs involved (Both ways) per month? **(Rs.)**				
C.2.l	Who paid for the travel expense? **(Refer COII Code Sheet - Table G)**				
C.2.m	If Others Specify				

C.3 OUT PATIENT CONTACTS

I would now like to know more about your use of outpatient and clinic based services for [Child's Name] in the last **6 months.** Please do not include any instances of accidents/ emergency and inpatient contacts. We will talk about it later.

Instruction – Read out whole list before recording response in Table 6 and Table 7

Table 5

Codes	Name
01	Community Doctor
02	Hospital Doctor
03	Pediatrician
04	Neurologist
05	Psychologist
06	Speech Therapist
07	Occupational Therapist
08	Physiotherapist
09	Homeopath
10	Ayurvedic
11	Others (Traditional healers, Religious healers, other branches of medicine)

Table 6

S. No		C.3.1	C.3.2	C.3.3	C.3.4	C.3.5
C.3.a	**Code (Refer to Table 5)**					
C.3.b	**Health Care Provider** (Details) *(If others Specify)*					
C.3.c	**Visits** **Probe:** How many visits did you make to this provider in the last 6 months?					
C.3.d	**Private** Service provider 1; **Government** Service Provider 2 *(use appropriate code)*					
C.3.e	**Accompanying person** **Probe:** What was the relationship of the accompanying adult(s) with [Child's Name]? **(Refer COII Code Sheet - Table A)**					
C.3.f	**Mode of Transport** **(Refer COII Code Sheet Table B)**					
C.3.g	**Travel Time** **Probe:** How much time did it take you to reach the service provider when going and coming back; per visit? **(Hours : Minutes)**	Going : / Coming Back :	Going : / Coming Back :	Going : / Coming Back :	Going : / Coming Back :	Going : / Coming Back :
C.3.h	**Travel Cost/ Distance** **Probe:** How much money did you spend on traveling both ways per visit? **Probe:** How far you had to travel to visit the care provider? (In case using private modes of transport and cannot estimate cost on fuel note distance) ***(Specify value Rs. OR kms)***	Going Rs. / Kms; Coming Back Rs. / Kms	Going Rs. / Kms; Coming Back Rs. / Kms	Going Rs. / Kms; Coming Back Rs. / Kms	Going Rs. / Kms; Coming Back Rs. / Kms	Going Rs. / Kms; Coming Back Rs. / Kms

C.3.i	**Waiting Time** **(Hours : Minutes)**<br>**Probe:** Waiting time to be seen by the provider?	:	:	:	:	:
C.3.j	**Time spent with the provider** **(Hours : Minutes)**	:	:	:	:	:
C.3.k	**Consultation fees paid per visit** **(Rs.)**<br>Instruction - Exclude any costs of tests or medicines					
C.3.l	**Who paid for the consultation?**<br>**(Refer COII Code Sheet - Table G)**					
C.3.m	**If consultation fees paid by others specify**					
C.3.n	**Food Expense** **(Rs.)**<br>**Probe:** Did you have to spend money on lunch/ dinner during this visit or food was bought at home from outside? What was the average amount spent per visit?					
C.3.o	How many times did you have to incur expenses on food while visiting the healthcare provider?					
C.3.p	**Comments**<br>(In case multiple healthcare providers visited in one visit)<br>(In case tests and medicine are included in the consultation costs and cannot be clearly differentiated.)					

Table 7

S. No		C.3.6	C.3.7	C.3.8	C.3.9	C.3.10
C.3.a	**Code (Refer to Table 5)**					
C.3.b	**Health Care Provider** (Details)<br>*(If others Specify)*					
C.3.c	**Visits**<br>**Probe:** How many visits did you make to this provider in the last 6 months?					
C.3.d	**Private** Service provider — 1<br>**Government** Service Provider — 2<br>*(use appropriate code)*					

C.3.e	**Accompanying person**<br>**Probe:** What was the relationship of the accompanying adult(s) with [Child's Name]?<br>**(Refer COII Code Sheet - Table A)**					
C.3.f	**Mode of Transport**<br>**(Refer COII Code Sheet Table B)**					
C.3.g	**Travel Time**<br>**Probe:** How much time did it take you to reach the service provider when going and coming back; per visit? **(Hours : Minutes)**	**Going** : **Coming Back** :	**Going** : **Coming Back** :	**Going** : **Coming Back** :	**Going** : **Coming Back** :	**Going** : **Coming Back** :
C.3.h	**Travel Cost/ Distance**<br>**Probe:** How much money did you spend on traveling both ways per visit?<br>**Probe:** How far you had to travel to visit the care provider? (In case using private modes of transport and cannot estimate cost on fuel note distance)<br>***(Specify value Rs. OR kms)***	**Going** Rs. / Kms<br>**Coming Back** Rs. / Kms	**Going** Rs. / Kms<br>**Coming Back** Rs. / Kms	**Going** Rs. / Kms<br>**Coming Back** Rs. / Kms	**Going** Rs. / Kms<br>**Coming Back** Rs. / Kms	**Going** Rs. / Kms<br>**Coming Back** Rs. / Kms
C.3.i	**Waiting Time** **(Hours : Minutes)**<br>**Probe:** Waiting time to be seen by the provider?	:	:	:	:	:
C.3.j	**Time spent with the provider** **(Hours : Minutes)**	:	:	:	:	:
C.3.k	**Consultation fees paid per visit** **(Rs.)**<br>Instruction - Exclude any costs of tests or medicines					
C.3.l	**Who paid for the consultation?**<br>**(Refer COII Code Sheet - Table G)**					
C.3.m	**If consultation fees paid by others specify**					
C.3.n	**Food Expense** **(Rs.)**<br>**Probe:** Did you have to spend money on lunch/ dinner during this visit or food was bought at home from outside? What was the average amount spent per visit?					

C.3.o	How many times did you have to incur expenses on food while visiting the healthcare provider?					
C.3.p	**Comments**<br>(In case multiple healthcare providers visited in one visit)<br>(In case tests and medicine are included in the consultation costs and cannot be clearly differentiated.)					

C.4 INPATIENT CONTACTS

Did [Child's Name] had to stay for a night/ multiple nights in a hospital/ nursing home in the last **6 months?**

Circle response	
YES	NO

Table 8	
**Code**	**Name**
1	Nursing home
2	Hospital

For **YES** refer Table 8– inquire further in the Table 9
If **NO** -> SKIP to **C.5**

Instruction - Note all follow-up visits related with inpatient contacts in section C.3 (Outpatient contacts)

Table 9				
**S. No**		**C.4.1**	**C.4.2**	**C.4.3**
**C.4.a**	**Code (Refer to Table 8)**			
**C.4.b**	**Private** Service provider — **1**<br>**Government** Service Provider — **2**<br>*(use appropriate code)*			
**C.4.c**	**Reason for admission**			
**C.4.d**	**Name of Hospital/ Nursing Home**			
**C.4.e**	**Accompanying person (Caretaker)**<br>**Probe:** What was the relationship of the accompanying adult(s) with the child? Exclude visitors.<br>**(Refer COII Code Sheet - Table A)**			

**C.4.f**	**Number of Nights**<br>**Probe**: How many nights did your child stay in the hospital/ nursing home?			
**C.4 g**	**Total Cost (Rs.)**<br>**Probe:** Total cost which may include doctor's fees, bed charges, medicines, ambulance charges, test's, travel costs and food costs			
**C.4.h**	**Comments**			

C.5 RELOCATION

In the last **6 months** did you have to relocate to another place of residence because of [Child's Name]?
Probe- To be closer to school or other supportive services.

Circle response	
YES	NO

If **NO** -> SKIP to **C.6**
If **YES** continue and explore further

S. No	Title	Total Cost (Rs)	Paid by (Refer COII Code Sheet - Table G)	If Others Specify
**C.5.1**	If **YES**, how much was your approx. expense?<br>**Probe:** Include shipping household goods, labor, brokerage, travel costs (site visits and shifting), looking at other properties			

C.6 EMERGENCY/ ACCIDENTS

In the last **6 months,** did you incur expenses on any emergencies or accidents for [Child's Name]?
Probe: Include the expenses incurred during time of emergency or accident i.e. doctor's fee, dressing charges, medicines, ambulance charges, travel cost, tests and food costs)

Circle response	
YES	NO

If **NO** -> SKIP to **C.7**
If **YES** continue, explore further in Table 10

Instruction - Record all follow-up visits related with accidents and emergency in section C.3 (Outpatient contacts)

Table 10

S. No	Description	Total Cost (Rs)	Paid for by (Refer COII Code Sheet - Table G)	If Others specify
C.6.1				
C.6.2				
C.6.3				

C.7 RELIGIOUS TRIPS\ RETREATS AND RITUALS

In the past **6 months,** have you gone to any place of worship or belief or organized any religious rituals specifically for [Child's Name]?

Probe: Some parents go to their hometown deities, Ajmer, Shirdi, Vaishno Devi, etc. Some go to local places like Nizamuddin Dargah, Sai Mandir, Hanuman Mandir and other places of belief.
Probe: Some parents organize religious rituals like feeding pundits, poor people, havans, and jaagran etc. They also donate in cash and kind at religious places for the betterment of their child's health.

Circle response	
YES	NO

If **NO** continue to **C.8**
If **YES** continue and explore further in Table 11

Instruction - Includes travel costs (back and forth), extra costs for accompanying person with the child, additional food expense and other organizational expenses.

Table 11

S. No		C.7.1	C.7.2	C.7.3	C.7.4
C.7.a	Name of Place/ Religious Practice				
C.7.b	Number of visits/ events/ nights				
C.7.c	Total cost (Rs)				
C.7.d	Who paid for the expenses? **(Refer COII Code Sheet - Table G)**				
C.7.e	If others specify				

C.8 INVESTIGATIONS (Not Admission Related)

I would now like to know whether in the last **6 months** did [Child's Name] had any of the following tests.

Circle response	
YES	NO

If **NO** -> SKIP to **C.9**
If **YES** continue, enter the sub category code and name explore in Table 13 and Table 14

Table 12

Codes	Name of Test
1	X rays
2	Blood tests
3	EEG
4	ECG
5	Ultrasound
6	Scans
7	Psychological test
8	Others (Specify)

Instruction - Include all costs associated with the tests – that is *the cost of test, travel cost (back and forth), extra costs for accompanying person with the child, additional food expense.*

Table 13						
S. No		C.8.1	C.8.2	C.8.3	C.8.4	C.8.5
C.8.a	Code **(Refer Table 12)**					
C.8.b	Name of Test (Description)<br>*If others specify*					
C.8.c	Government Lab 1<br>Private Lab 2<br>Others* 3					
C.8.d	*If others specify*					
C.8.e	Total Cost **(Rs)**					
C.8.f	Who paid the expenses?<br>**(Refer COII Code Sheet - Table G)**					
C.8.g	*If others specify*					

**Includes Non-Governmental Organizations, Charitable Trusts, Free camps, CSR activities*

Table 14						
S. No		C.8.6	C.8.7	C.8.8	C.8.9	C.8.10
C.8.a	Code **(Refer Table 12)**					
C.8.b	Name of Test (Description)<br>*If others specify*					
C.8.c	Government Lab 1<br>Private Lab 2<br>Others* 3					
C.8.d	*If others specify*					
C.8.e	Total Cost **(Rs)**					
C.8.f	Who paid the expenses?<br>**(Refer COII Code Sheet - Table G)**					
C.8.g	*If others specify*					

C.9 COMPLIMENTARY MEDICATIONS (NOT ADMSSION RELATED)

I would like to know if in the last **6 months** [Child's Name] has taken any medicines which are supplementary, ayurvedic, homeopathic or traditional in nature?

Circle response	
YES	NO

If **NO** -> SKIP to **C.10**
If **YES** continue and explore further in Table 15 & Table 16 below

Instruction - Use codes wherever necessary

Table 15						
Q. No	S. No	C.9.1	C.9.2	C.9.3	C.9.4	C.9.5
C.9.a	Name of Drug					
C.9.b	Brand					
C.9.c	Used to Treat<br>**(Refer COII Code Sheet - Table D)**					
C.9.d	*If any other specify*					
C.9.e	Free medication 1<br>Paid medication 2<br>*(use appropriate code)*					
C.9.f	Dosage/ Units<br>**(Refer COII Code Sheet - Table E)**					
C.9.g	Value of Dose					
C.9.h	Frequency<br>**(Refer COII Code Sheet - Table F)**					
C.9.i	*If other type of frequency specify*					
C.9.j	Number of days prescribed					
C.9.k	Cost per bottle/ strip/ pack					
C.9.l	Number of bottle/ strip/ pack used in last 6 months					
C.9.m	Total cost incurred in the last 6 months **(Rs)**					

		Table 16				
Q. No	S. No	C.9.6	C.9.7	C.9.8	C.9.9	C.9.10
C.9.a	Name of Drug					
C.9.b	Brand					
C.9.c	Used to Treat **(Refer COII Code Sheet - Table D)**	☐	☐	☐	☐	☐
C.9.d	*If any other specify*					
C.9.e	Free medication 1<br>Paid medication 2<br>*(use appropriate code)*	☐	☐	☐	☐	☐
C.9.f	Dosage/ Units **(Refer COII Code Sheet - Table E)**	☐	☐	☐	☐	☐
C.9.g	Value of Dose					
C.9.h	Frequency **(Refer COII Code Sheet - Table F)**	☐	☐	☐	☐	☐
C.9.i	*If other type of frequency specify*					
C.9.j	Number of days prescribed					
C.9.k	Cost per bottle/ strip/ pack					
C.9.l	Number of bottle/ strip/ pack used in last 6 months					
C.9.m	Total cost incurred in the last 6 months **(Rs)**					

C.10 MEDICATION (NOT ADMISSION RELATED)

I would like to know if in the last **6 months** [Child's Name] has taken any allopathic medicines.

Circle response	
YES	NO

If **NO** -> SKIP to **C.11**
If **YES** continue and explore further in Table 17 & Table 18 below

Instruction - Use codes wherever necessary

		Table 17				
Q. No	S. No	C.10.1	C.10.2	C.10.3	C.10.4	C.10.5
C.10.a	Name of Drug					
C.10.b	Brand					
C.10.c	Used to Treat **(Refer COII Code Sheet - Table D)**	☐	☐	☐	☐	☐
C.10.d	*If any other specify*					
C.10.e	Free medication 1<br>Paid medication 2<br>*(use appropriate code)*	☐	☐	☐	☐	☐
C.10.f	Dosage/ Units **(Refer COII Code Sheet - Table E)**	☐	☐	☐	☐	☐
C.10.g	Value of Dose					
C.10.h	Frequency **(Refer COII Code Sheet - Table F)**	☐	☐	☐	☐	☐
C.10.i	*If other type of frequency specify*					
C.10.j	Number of days prescribed					
C.10.k	Cost per bottle/ strip/ pack					
C.10.l	Number of bottle/ strip/ pack used in last 6 months					
C.10.m	Total cost incurred in the last 6 months **(Rs)**					

		Table 18				
Q. No	S. No	C.10.6	C.10.7	C.10.8	C.10.9	C.10.10
C.10.a	Name of Drug					
C.10.b	Brand					
C.10.c	Used to Treat **(Refer COII Code Sheet - Table D)**	☐	☐	☐	☐	☐

C.10.d	*If any other specify*					
C.10.e	Free medication 1<br>Paid medication 2<br>*(use appropriate code)*	☐	☐	☐	☐	☐
C.10.f	Dosage/ Units<br>**(Refer COII Code Sheet - Table E)**	☐	☐	☐	☐	☐
C.10.g	Value of Dose					
C.10.h	Frequency<br>**(Refer COII Code Sheet - Table F)**	☐	☐	☐	☐	☐
C.10.i	*If other type of frequency specify*					
C.10.j	Number of days prescribed					
C.10.k	Cost per bottle/ strip/ pack					
C.10.l	Number of bottle/ strip/ pack used in last 6 months					
C.10.m	Total cost incurred in the last 6 months **(Rs)**					

C.11 EQUIPMENT RECOMMENDED BY SPECIALIST

In the last **6 months,** have you purchased any equipment/ toy/ manual recommended by a specialist for [Child's Name] OR had made any major adaptations at your home for [Child's Name]?

Probe: If the participant has referred to any online resource/ YouTube video was it from a specialist.

**Circle response**	
YES	NO

If **NO** -> SKIP to **C.12**
If **YES** continue and explore further in Table 19 & Table 20

Table 19

S. No		C.11.1	C.11.2	C.11.3	C.11.4	C.11.5
C.11.a	Equipment/ Measures					
C.11.b	Estimated Cost **(Rs)**					
C.11.c	Paid for by<br>**(Refer COII Code Sheet - Table G)**	☐	☐	☐	☐	☐
C.11.d	*If others specify*					
C.11.e	Recommended by/ source of information<br>**(Refer COII Code Sheet - Table C)**	☐	☐	☐	☐	☐
C.11.f	*If any other source specify*					

Table 20

S. No		C.11.6	C.11.7	C.11.8	C.11.9	C.11.10
C.11.a	Equipment/ Measures					
C.11.b	Estimated Cost **(Rs)**					
C.11.c	Paid for by<br>**(Refer COII Code Sheet - Table G)**	☐	☐	☐	☐	☐
C.11.d	*If others specify*					
C.11.e	Recommended by/ source of information<br>**(Refer COII Code Sheet - Table C)**	☐	☐	☐	☐	☐
C.11.f	*If any other source specify*					

C.12 WORKSHOPS AND TRAININGS

In the last **6 months** had there been any extra costs with workshops, trainings or conferences related with [Child's Name] autism?

Circle response	
YES	NO

If **NO** -> SKIP to **C.13**
If **YES** continue and explore further in Table 21

Table 21

S. No		C.12.1	C.12.2	C.12.3	C.12.4	C.12.5
C.12.a	Workshop/ Training/ Conference					
C.12.b	Time spent for the workshop (Include travel) **(Hours : Minutes)**	☐☐ : ☐☐	☐☐ : ☐☐	☐☐ : ☐☐	☐☐ : ☐☐	☐☐ : ☐☐
C.12.c	Estimated Cost (Include travel) **(Rs.)**					
C.12.d	Paid for by **(Refer COII Code Sheet - Table G)**	☐	☐	☐	☐	☐
C.12.e	*If others specify*					
C.12.f	Recommended by/ source of information **(Refer COII Code Sheet – Table C)**	☐	☐	☐	☐	☐
C.12.g	*If any other source specify*					

C.13 SPECIAL DIET

In the last **6 months** have you given [Child's Name] any special diet?

Circle response	
YES	NO

If **NO** -> SKIP to **C.14**
If **YES** continue and explore further in Table 22

Table 22

S. No		C.13.1	C.13.2	C.13.3
C.13.a	Description			
C.13.b	Recommended by **(Refer COII Code Sheet - Table C)**	☐	☐	☐
C.13.c	*If any other source specify*			
C.13.d	Estimated Costs (Last 6 months) **(Rs.)**			
C.13.e	Paid By **(Refer COII Code Sheet - Table G)**	☐	☐	☐
C.13.f	*If Others specify*			

C.14 SUPPORT AND CARE (INCLUDES ADMISSION RELATED)

In the last **6 months,** have you or any family member or a friend had to stop or reduce their regular work or activities due to [Child's Name] Autism?

Circle response	
YES	NO

If **NO** -> SKIP to **C.15**
If **YES** continue and explore further in Table 23

Table 23

S. No		C.14.1	C.14.2	C.14.3	C.14.4
C.14.a	Caretaker				
C.14.b	Relationship with the child **(Refer COII Code Sheet - Table A)**	☐	☐	☐	☐
C.14.c	No. of days of work reduced in last 6 months **Probe:** Exclude sanctioned holidays				
C.14.d	Type of work reduced or stopped Probe: Exclude holidays				
C.14.e	If Applicable (Income lost per day) **(Rs)**				
C.14.f	No. of paid leaves				
C.14.g	No. of unpaid leaves				

C.15 COST OF CERTIFICATION

Did you incur any expenses getting specific certifications for [Child's Name]? **(It can be before 6 months)**

Probe: Details of the certificate and estimated cost incurred in a acquiring it (travel, registration or any other expense)

Circle response	
YES	NO

If **NO** -> SKIP to **C.16**
If **YES** continue and explore further

Table 24

S. No	Description	Total Cost (Rs)	Paid for by (Refer COII Code Sheet - Table G)	If Others Specify
C.15.1				
C.15.2				

C.16 OCCUPATIONAL ADJUSTMENTS

Would you or any of your family members would have returned to work if [Child's Name] did not have Autism?

Circle response	
YES	NO

If **NO** skip to **C.17**
If **YES** continue and explore further in Table 24

Table 25

S. No		C.16.1	C.16.2
C.16.a	Relationship with [Child's name] **(Refer COII Code Sheet - Table A)**		
C.16.b	Last Job title		
C.16.c	When did you stop working? **(Year)**		
C.16.d	Salary last drawn (per month) **(Rs)**		

C.17 GOVERNMENT REBATES/ SCHEMES

Have you availed any rebates/ schemes from the govt. for children with autism for [Child's Name] in the last **6 months**?

Probe: Income tax rebates, schemes by Govt. for children with Autism or any other.

Circle response	
YES	NO

If **NO** End Interview with script.
If **YES** continue and explore further in Table 25

Table 26

S. No	Description	Total Value (Rs)
C.17.1		
C.17.2		

End Script
Thank you for your time. The information provided was very helpful.

NOTES

Cost of Illness Inventory (COII)

Code Sheet

Table A	
**Codes**	**Relationships**
01	Father
02	Mother
03	Brother
04	Sister
05	Grand Mother
06	Grand Father
07	Friend (Uncle)
08	Friend (Aunty)
09	Relative (Uncle)
10	Relative (Aunt)
11	Neighbour
12	Others

Table B	
**Codes**	**Transports**
01	Local Bus
02	Metro
03	Local Train
04	Hired Cab
05	Auto Rickshaw
06	Cycle Rickshaw
07	Own vehicle (4 wheeler)
08	Own vehicle (2 wheeler)
09	Own vehicle (4 wheeler CNG)
10	Neighbours Vehicle
11	Employer Provided Car
12	Walking
13	Others

Table C	
**Codes**	**Specialists**
01	Community Doctor
02	Hospital Doctor
03	Pediatrician
04	Neurologist
05	Psychologist
06	Speech Therapist
07	Occupational Therapist
08	Physiotherapist
09	Homeopath
10	Ayurvedic
11	Special Educator
12	School Teacher
13	Others (Traditional healers, Religious healers, other branches of medicine)

Table D	
Codes	Used to treat options
01	Asthma
02	Eczema/ other skin condition
03	Allergies/ hay fever
04	Anemia
05	Diabetes
06	Epilepsy
07	Constipation/ other bowel problems
08	Sleep problems
09	Anxiety/ depression
10	Hyperactivity/ inattention
11	Behavioral problems, e.g. irritability, aggression, self-injury
12	Dietary and Nutritional Supplements
13	Others (Specify)

Table E	
Codes	Dosage/ Units
01	Milligram
02	Application
03	Capsule
04	Drop
05	Gram
06	Inhaled
07	Liter
08	Microgram
09	Puff
10	Suppository
11	Tablespoon
12	Tablet
13	Teaspoon
14	Trans – dermal (patch)
15	International Units
16	Globules

Table F	
Codes	Frequency
01	Once daily
02	Twice Daily
03	Three times daily
04	Four times daily
05	Alternate days
06	Once a week
07	Twice a week
08	Once a month
09	Twice a month
10	As required
11	Continuous
12	Others (please specify)

Table G	
Codes	Relationships
01	Father
02	Mother
03	Brother
04	Sister
05	Grand Mother
06	Grand Father
07	Friend (Uncle)
08	Friend (Aunty)
09	Relative (Uncle)
10	Relative (Aunt)
11	Neighbour
12	Employer (Reimbursed)
13	Insurance (Reimbursed)
14	Others (Specify)

Section B: Respondent Information		Variable Name
B.1 Visit	1. Baseline (Enrolment)<br>2. End line (9M) (Post-allocation)<br>3. Follow-up (15M) (Close-out)	coii_visit
B.2. Interactional partner the same as at baseline (Only applicable at endline)	1. Yes<br>2. No	coii_samerepondentbaseline
B.3. New respondent since baseline (Only applicable at endline) (Name of Respondent – Relationship with child)		coii_newrespondentbaseline
B.4. Interactional partner the same as at 9 months (Only applicable at follow-up)	1. Yes<br>2. No	coii_samerepondent9m
B.5. New respondent since 9 months (Only applicable at follow-up) (Name of Respondent – Relationship with child)		coii_newrespondent9m
